# Correction: Current and future costs of cancer attributable to insufficient leisure-time physical activity in Brazil

**DOI:** 10.1371/journal.pone.0293771

**Published:** 2023-10-26

**Authors:** Ronaldo Corrêa Ferreira da Silva, Thainá Alves Malhão, Leandro F. M. Rezende, Rafael da Silva Barbosa, Arthur Orlando Correa Schilithz, Luciana Grucci Maya Moreira, Paula Aballo Nunes Machado, Fabio Fortunato Brasil de Carvalho, Maria Eduarda Leão Diogenes

There are errors in the Funding section. The correct Funding statement is: This research received financial support from Climate and Land Use Alliance (CLUA) (Grant Number G-2007-56990) and Brazilian National Cancer Institute José Alencar Gomes da Silva (INCA). https://www.climateandlandusealliance.org/ www.inca.gov.br The funder had no role in study design, data collection and analysis, decision to publish, or preparation of the manuscript and does not necessarily share the positions expressed in the Grantee’s publication.

We thank the Public Labour Prosecution Office (Ministério Público do Trabalho—MPT) for paying the publication fees through public civil action No. 0100263–41.2021.5.01.0005.
