# Supplementary material for: Current and future costs of cancer attributable to insufficient leisure-time physical activity in Brazil
Source: PLoS One. 2023 Jul 10;18(7):e0287224. doi: 10.1371/journal.pone.0287224 (PMC10332606; doi:10.1371/journal.pone.0287224)
Supplement: S1 Appendix — (DOC) [file pone.0287224.s001.doc]

**Supporting Information**

**Current and future costs of cancer attributable to insufficient leisure-time physical activity in Brazil**

Ronaldo Corrêa Ferreira da Silva, Thainá Alves Malhão, Leandro F. M. Rezende, Rafael da Silva Barbosa, Arthur Orlando Correa Schilithz, Luciana Grucci Maya Moreira, Paula Aballo Nunes Machado, Fabio Fortunato Brasil de Carvalho, Maria Eduarda Leão Diogenes

**S1 File. Meta-analysis from World Cancer Research Fund (WCRF) and the American Institute for Cancer Research (AICR).**

| **Cancer type** | **Reference** |
| --- | --- |
| Breast | World Cancer Research Fund/ American Institute for Cancer Research/ Imperial College London. Continuous Update Project Team Members. World Cancer Research Fund International Systematic Literature Review. The Associations between Food, Nutrition and Physical Activity and the Risk of Breast Cancer, Jan. 2017. Available at https://www.wcrf.org/dietandcancer |
| Colon | World Cancer Research Fund/ American Institute for Cancer Research/ Imperial College London. Continuous Update Project Team Members. World Cancer Research Fund International Systematic Literature Review. The Associations between Food, Nutrition and Physical Activity and the Risk of Colorectal Cancer Sep. 2017. Available at https://www.wcrf.org/dietandcancer |
| Endometrium | World Cancer Research Fund/ American Institute for Cancer Research/ Imperial College London. Continuous Update Project Team Members. World Cancer Research Fund International Systematic Literature Review. The Associations between Food, Nutrition and Physical Activity and the Risk of Endometrial Cancer, Dec. 2012. Available at https://www.wcrf.org/dietandcancer |

**S2 File.** **Summary of the data considered in the macrosimulation model of current and future federal direct healthcare costs of cancer in the Brazilian Public Health System attributable to insufficient leisure-time physical activity.**

| **Parameter** | **Variable** | **Commentary** | **Source/ Year** |
| --- | --- | --- | --- |
| Insufficient leisure-time physical activity. | Prevalence data and median of insufficient leisure-time physical activity in adults ≥ 20 years who rely exclusively on the public health system. | We calculated the variable “MET-hour-week”, multiplying the frequency of leisure-time physical activity by the duration in hours and by the MET of the modality.  We considered insufficient leisure- time physical activity when MET-hour-week was < 7.5.  We stratified by sex and exposition category.  Exposition categories: ≥ 7.5 MET-hour-week; < 7.5 MET-hour-week. | National Household Sample Survey (PNAD 2008) and National Health Survey (PNS 2019). |
| Relative risk. | Relative risk. | We used the highest vs lowest relative risks obtained from meta-analysis. We stratified by sex, when available, cancer type, and exposition category.  Exposition categories: ≥ 7.5 MET-hour-week; < 7.5 MET-hour-week. | WCRF/AICR systematic review reports. |
| Direct healthcare cost. | Values of inpatient procedures related to cancer in adults ≥ 30 years paid by the federal government. | Federal direct healthcare costs of inpatient procedures related to cancer approved for payment in the Brazilian Unified Health System. We stratified by sex and cancer type. | Hospital Information System of the Brazilian Public Health System  (SIH-SUS)  2008-2019. |
| Direct healthcare cost. | Values of outpatient procedures related to cancer in adults ≥ 30 years paid by the federal government. | Federal direct healthcare costs of outpatient procedures related to cancer approved for payment in the Brazilian Unified Health System. We stratified by sex and cancer type. | Ambulatory Information System of the Brazilian Public Health System  (SIA-SUS) 2008-2019. |

**S3 File. Hyperlinks to publicly archived datasets.**

**PNAD 2008 –** <https://www.ibge.gov.br/estatisticas/sociais/saude/19898-suplementos-pnad3.html?=&t=microdados>

**PNS 2019 -** <https://www.ibge.gov.br/estatisticas/sociais/saude/9160-pesquisa-nacional-de-saude.html?=&t=microdados>

**SIA-SUS -** <ftp://ftp.datasus.gov.br/dissemin/publicos/siasus/200801_/dados>

**SIH-SUS -** <ftp://ftp.datasus.gov.br/dissemin/publicos/sihsus/200801_/dados> 

**S4 File. Methodological differences between the surveys.**

1. While the PNAD evaluated the practice of physical activity in the last three months, the PNS evaluated it in the twelve months.
2. The response options for the variable “number of days per week that practice physical activity” were different (PNAD: from 1 to 2, from 3 to 4, from 5 to 6, every day; PNS: values from 1 to 7). For constructing the variable “MET-hours per week” in the PNAD, we considered the lower cut-off point of the available categories.
3. The response options for the variable “duration of physical activity on the day it is practiced” were different (PNAD: less than 20 minutes, from 20 to 29 minutes, 30 minutes or more; PNS: number of hours and minutes). For constructing the variable “MET-hours per week” in the PNAD, we considered the midpoint in the category "less than 20 minutes" and the lower cut-off point in the other categories. To create the variable “duration in hours”, we divided the value found in the variable “duration in minutes” by 60.
4. The response options for the variable “main modality of leisure-time physical activity” and the MET considered for the construction of the variable “MET-hours per week” also differed (PNAD: Walking = 3.5 MET; Soccer, basketball, gymnastics aerobics, running or tennis = 7 MET; Others = 3.5 MET; No longer practicing = 0 MET; PNS: Walking = 3.5 MET, Treadmill walking: 3.5 MET; Running/ jogging = 8.3; Treadmill running = 8.3; Weight training = 3.5; Aerobics/ spinning/ step/ jump = 7.8; Water aerobics = 5.5; Gymnastics/ localized/ Pilates/ stretching/ yoga = 3.5; Swimming = 5.8; Martial arts and fighting = 7.8; Bicycle/ exercise bike = 4.8; Soccer = 7; Basketball = 6.5; Volleyball = 3; Tennis = 7.3; Dance (with the aim of practicing physical activity) = 3; Others = 3.5).

**S5 File. Cancer types and 10th revision of the International Statistical Classification of Diseases and Related Health Problems (ICD-10) codes included in the study.**

| **Cancer type** | **ICD-10 code** |
| --- | --- |
| Breast | C50, C50.0, C50.1, C50.2, C50.3, C50.4, C50.5, C50.6, C50.8, C50.9 |
| Colon | C18, C18.0, C18.1, C18.2, C18.3, C18.4, C18.5, C18.6, C18.7, C18.8, C18.9 |
| Colorectal | C18, C18.0, C18.1, C18.2, C18.3, C18.4, C18.5, C18.6, C18.7, C18.8, C18.9, C19, C20 |
| Endometrium | C54, C54.0, C54.1, C54.2, C54.3, C54.8, C54.9 |
| All invasive cancers | C00-C97 |

## 
